# MicroRNA-202 inhibits cell proliferation, migration and invasion of glioma by directly targeting metadherin

**DOI:** 10.3892/or.2022.8344

**Published:** 2022-06-06

**Authors:** Jinsheng Yang, Bo Fan, Yachao Zhao, Junchao Fang

Oncol Rep 18: 1670–1678, 2017; DOI: 10.3892/or.2017.5815

Following the publication of this paper, it was drawn to the Editors' attention by a concerned reader that certain of the panels shown for the cell invasion assays in Figs. 2C and 6C were strikingly similar to data appearing in different form in other articles by different authors. Owing to the fact that the contentious data in the above article had were already under consideration for publication prior to its submission to *Oncology Reports*, the Editor has decided that this paper should be retracted from the Journal. The authors were asked for an explanation to account for these concerns, but the Editorial Office did not receive a reply. The Editor apologizes to the readership for any inconvenience caused.

